# A Case of Subungual Exostosis Mimicking Verruca Vulgaris

**DOI:** 10.7759/cureus.76564

**Published:** 2024-12-29

**Authors:** Fiona S Gruzmark, Lacey Zimmerman, Joel Joyce

**Affiliations:** 1 Department of Dermatology, University of Illinois, Chicago, USA; 2 Department of Medicine, Division of Dermatology, NorthShore University HealthSystem, Evanston, USA

**Keywords:** dermatology, exostoses, onycholysis, pediatrics, warts

## Abstract

Subungual exostosis is a benign bone tumor causing nail deformities and possibly pain for the patient. Etiology includes trauma, infection, and activation of a cartilaginous cyst, more commonly seen in adult patients. Here, we present a case of subungual exostosis in a pediatric patient that initially mimicked subungual verruca vulgaris. After failed cryotherapy, surgical management allowed for symptom resolution without recurrence. This case highlights the need to consider subungual exostosis in the differential when managing suspected verruca vulgaris associated with the nail unit, a common finding in pediatric patients, particularly if the lesions are refractory to traditional verruca treatments.

## Introduction

Subungual exostosis (SE) is a relatively uncommon benign bone tumor. Although the exact pathogenesis and prevalence of the disease are unclear, it is thought to be a reactive process secondary to microtrauma [1], with infection, tumors, and hereditary abnormalities being involved in the etiopathogenesis [2]. Clinically, this condition presents as a firm nodule with a hyperkeratotic smooth surface on the nail plate with associated pain and erythema. The average age of disease onset has been reported to be 26 years, more commonly on the great toe and with a female-to-male ratio of 1:1 [1]. There is a wide differential diagnosis to be considered, such as onychomatricoma, keratoacanthoma, subungual epidermoid inclusions, and a subungual verruca [2]. 

The bone tumor is formed from fibrous tissue, with its cartilaginous cap being composed of fibrocartilage [1]. On histopathology, there is bone deposition in the mid and lower dermis, with mature bone tissue with trabeculae [2]. These lesions typically cause pain, erythema, and a deformed nail bed, which worsens quality of life. Given the relative rarity of this diagnosis and common misdiagnosis, it is crucial to better elucidate its presentation and management [1]. Differentiating SE from other common periungual lesions, such as verruca vulgaris (VV), can be challenging and often leads to delayed or misdirected management. Here, we present a pediatric patient who was diagnosed and treated for SE, while the initial presentation of the disease mimicked a subungual VV. 

## Case presentation

**Figure 1:**
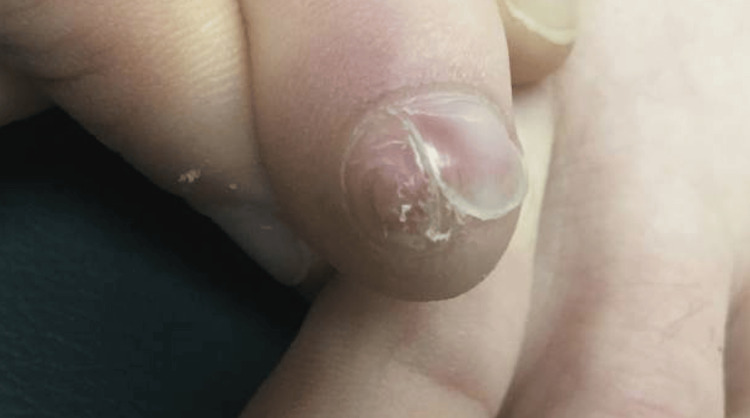
Erythematous, firm nodule on the lateral nail bed of the fifth toe with absence of overlying scale and adjacent separation of the nail plate from the bed

A 14-year-old female patient presented to dermatology with an erythematous, firm, scaled nodule on the lateral, distal nail bed of the right fifth toe, with breakage and fracturing of the adjacent nail plate, for over one year. It was painful with activity. She had a known history of plantar warts and had previously undergone cryotherapy treatment of these with success. Therefore, a presumptive diagnosis of subungual verruca was made, and it was treated with cryotherapy. Two months later, persistence of the nodule was noted, but with relative absence of scale (Figure 1).

**Figure 2:**
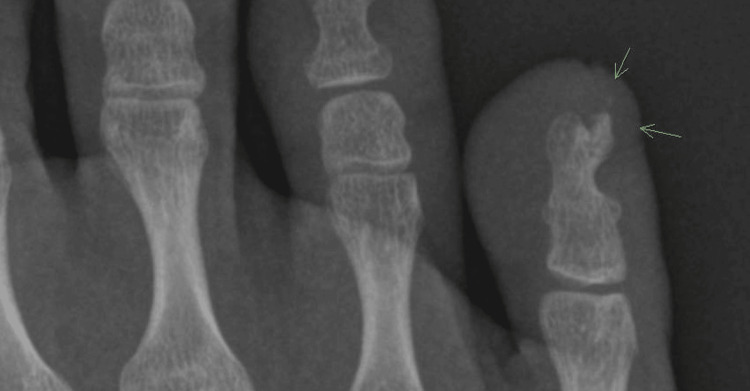
X-ray showing a radio-opaque mass consistent with a subungual exostosis. Arrow denotes the subungual exostosis arising from the distal phalanx

Given the lack of treatment response and change in presentation, SE was considered, among other neoplasms. An X-ray of the foot demonstrated a radio-opaque mass on the distal phalanx, suggestive of a SE (Figure 2).

The patient was referred to orthopedic surgery, and the nodule was excised with histopathologic confirmation of SE and symptom resolution.

## Discussion

The most common cause of a scaly nodule with disruption of the nail plate is a VV, which typically presents as a flesh-colored papule with a gray-white or brown overlying scale. Its peak incidence is in late childhood and adolescence [3]. Ungual warts, which are most commonly caused by human papillomavirus (HPV) types 1, 2, 4, 27, and 57 in children and young adults, present as nodular lesions that may separate the nail plate from the bed [4], resembling our patient's presentation. However, given the lack of treatment response and the atypical presentation upon return, other diagnoses, including SE, were considered. 

The majority of SE cases are seen in the second and third decades of life, and 70% of cases appear in the nail bed of the first toe [5], which was inconsistent with our patient's presentation. However, the presence of a firm and painful nodule in the nail bed that did not respond to typical treatment for VV was suggestive of SE [6]. Other causes of nodular growths of the nail bed, including other tumors of the bone, cartilage, or soft tissues and subungual melanoma [5], were considered, but excluded, based on imaging and ultimately histopathology. 

The etiology of SE includes trauma, infection, and cartilage cyst activation [2,5]. SE is a benign process; however, it has been reported to have COL12A1 and COL4A5 rearrangements and a t(X;6) translocation [2], suggesting that it is an abnormal, neoplastic growth. Histopathologic and radiologic examinations are used for diagnosis, with dermoscopy being a useful adjunct [7]. As in this patient, mature lesions on X-ray appear as radio-opaque calcified and trabecular bone that is contiguous with the cortex of the distal phalanx [5] (Figure 2). Histopathology reveals a trabecular bone pattern, sometimes with a fibrocartilaginous cap [2,6]. Dermoscopy may include features of vascular ectasia, ulceration, hyperkeratosis, and/or onycholysis [6]. The preferred treatment is surgical excision [6,7].

## Conclusions

We presented a case of SE mimicking subungual VV in a pediatric patient. SE is commonly misdiagnosed as a subungual VV, as both lesions may present as hyperkeratotic papules or nodules on the nail plate, but may be differentiated based on treatment response, imaging, and histopathology. While SE has been well-reported in adult populations, it is less commonly seen in pediatric patients and may be commonly misdiagnosed. Although this is a benign bone tumor, it does cause pain and nail deformities for patients, necessitating prompt diagnosis and management. It is essential to consider SE as a differential when managing VV associated with the nail unit, a common finding in pediatric patients, particularly if the lesions are refractory to traditional VV treatments.

## References

[1] DaCambra MP, Gupta SK, Ferri-de-Barros F (2014). Subungual exostosis of the toes: a systematic review. Clin Orthop Relat Res.

[2] Das PC, Hassan S, Kumar P (2019). Subungual exostosis - clinical, radiological, and histological findings. Indian Dermatol Online J.

[3] Plasencia JM (2000). Cutaneous warts: diagnosis and treatment. Prim Care.

[4] Herschthal J, McLeod MP, Zaiac M (2012). Management of ungual warts. Dermatol Ther.

[5] Olvi LG, Gonzalez ML, da Cunha IW, Santini-Araujo E, Kalil RK (2020). Subungueal exostosis. Tumors and Tumor-Like Lesions of Bone.

[6] Zhang W, Gu L, Fan H, Shen X, Lu H (2020). Subungual exostosis with an unusual dermoscopic feature. JAAD Case Rep.

[7] Li H, Li H, Qi X (2022). Clinical diagnosis and treatment of subungual exostosis in children. Front Pediatr.

